# Recent advances in omics-based research of mitral valve disease

**DOI:** 10.3389/fcvm.2026.1789634

**Published:** 2026-05-15

**Authors:** Shilin Jin, Shishi Wu, Di Zhang, Yantao Luo, Nabila Bouatia-Naji, Mengyao Yu

**Affiliations:** 1 Human Phenome Institute, Shanghai Pudong Hospital, Fudan University Pudong Medical Centre, Fudan University, Shanghai, China; 2 Inserm, PARCC, Université Paris Cité, Paris, France

**Keywords:** epigenomics, genomics, mitral valve disease, multi-omics, omics, proteomics, transcriptomics

## Abstract

Mitral valve disease (MVD), particularly mitral valve prolapse (MVP), is a common heart valve condition affecting millions of people worldwide. Its pathological mechanism is complex, involving multiple factors such as extracellular matrix remodeling, inflammatory responses, and genetic susceptibility. In recent years, with the development of high-throughput sequencing and analysis technologies, omics approaches including genomics, epigenomics, transcriptomics, proteomics, metabolomics and multi-omics integration have been widely used in MVD research. These technologies have greatly deepened our understanding of the molecular basis of the disease. This review systematically organizes the scientific literature from the past decade on the use of omics technologies to study mitral valve disease, especially MVP and myxomatous mitral valve disease (MMVD). We classify the findings into genomics, epigenomics, transcriptomics, proteomics, metabolomics and multi-omics analysis. We summarize the core discoveries in each area and discuss future research directions, with the goal to provide a theoretical basis for developing new diagnostic markers and targeted therapeutic strategies.

## Introduction

Mitral valve disease (MVD) is the most common form of heart valve disease, affecting more than 10% of individuals over 75 years old and expected to increase further as life expectancy extends (1). Mitral valve disease comprises a heterogeneous group of disorders affecting the mitral valve. These include primary conditions such as mitral valve prolapse (MVP), functional mitral regurgitation (MR) resulting from left ventricular remodeling, and mitral stenosis (MS), most commonly of rheumatic origin. MVP is characterized by leaflet elongation and redundancy, leading to incomplete closure and regurgitation. MVP can be further classified into Barlow's disease (BD) and fibroelastic deficiency (FED), which display distinct clinical and molecular features (2). Importantly, MVP can occur in both non-syndromic and syndromic forms. Syndromic MVP is frequently observed in connective tissue disorders such as Marfan syndrome, and other inherited conditions affecting extracellular matrix integrity, where valvular abnormalities represent part of a broader systemic phenotype (3). MR results from failure of proper leaflet coaptation, causing backward blood flow (4), whereas MS is defined by narrowing of the mitral valve orifice (5). In recent decades, the major causes of mitral valve dysfunction have shifted from rheumatic heart disease to degenerative conditions, particularly MVP and MR, which are the primary focus of most recent omics studies. Although surgery (such as valve repair or replacement) is the main treatment for severe MVD, it cannot stop the early progression of the disease. There is also a lack of effective drug therapies to slow or reverse the degenerative changes in the valve (6, 7). Therefore, it is crucial to deeply investigate the pathogenesis of MVD and find new diagnostic and therapeutic targets.

The rise of omics technologies has provided an unprecedented opportunity to systematically reveal the molecular networks of complex diseases (8). Through comprehensive analysis of multiple molecular levels, including DNA (genomics), epigenetic modifications (epigenomics), RNA transcripts (transcriptomics), proteins (proteomics), and metabolic profiling (metabolomics), researchers can map the complete pathophysiological landscape of MVD from different perspectives. In particular, multi-omics integrated analysis can link data from different levels to build a causal chain from genetic variation to clinical phenotype.

Despite the rapid proliferation of high-throughput datasets in cardiovascular research, the molecular landscape of mitral valve disease (MVD) remains fragmented across isolated studies, which limited integration across different omics layers. Given the urgent need for non-surgical therapeutic strategies and the growing shift toward precision medicine, a comprehensive and integrative synthesis of omics findings is both timely and necessary.

**Figure 1:**
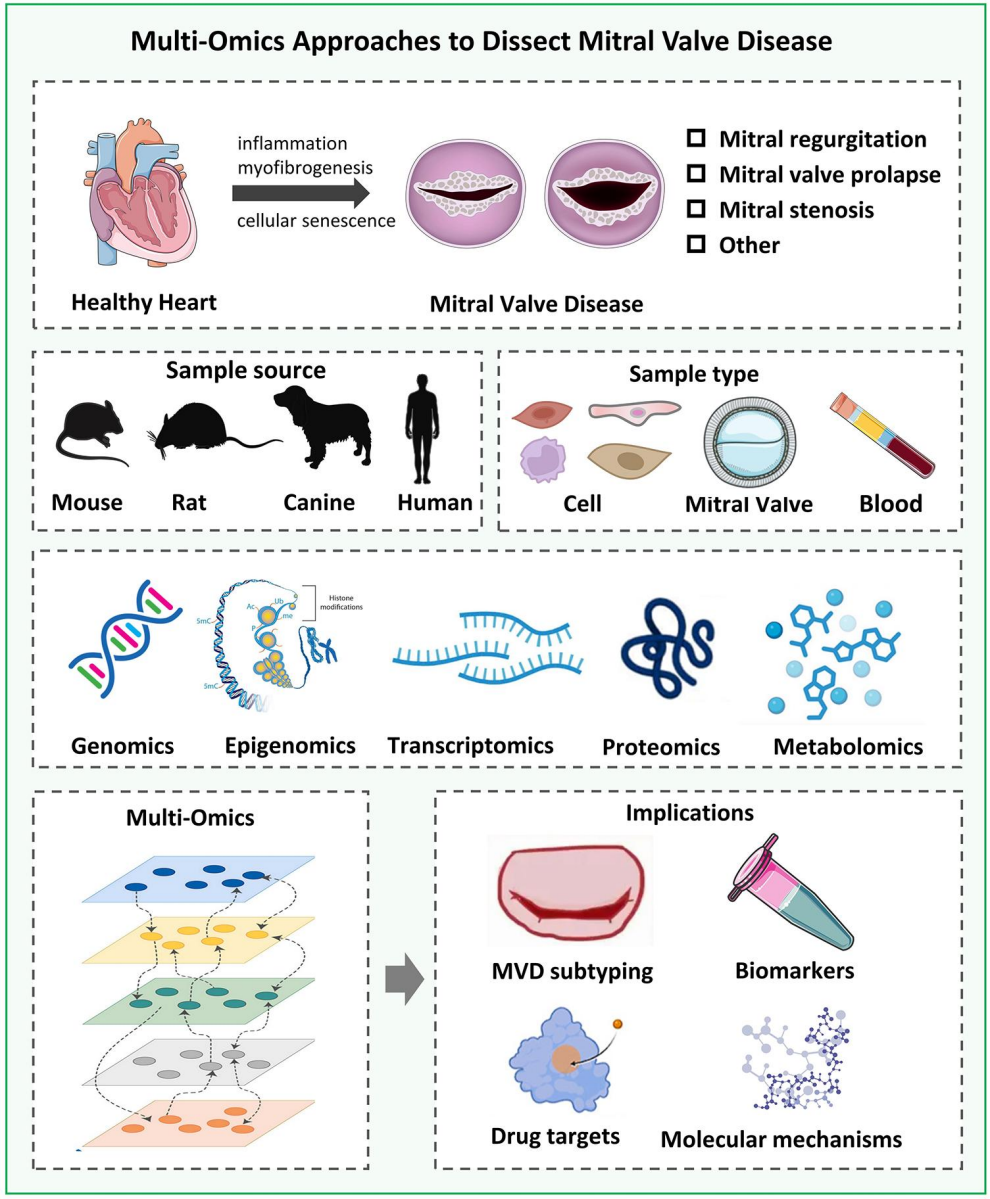
Multi-Omics approaches to dissect mitral valve disease. The schematic illustrates how genomics, epigenomics, transcriptomics, proteomics, and metabolomics interact across molecular layers to link genetic variation to cellular phenotypes and tissue remodeling, ultimately contributing to disease progression. The figure highlights the shift from single-layer analyses to integrated multi-omics interpretation, enabling a more comprehensive understanding of disease mechanisms and the identification of potential biomarkers and therapeutic targets.

In this review, we outline the transition in valvular heart disease research from single- to multi-omics approaches, with a particular emphasis on how these approaches collectively contribute to understanding disease mechanisms. We also discuss how this integrated perspective moves the field beyond isolated observations toward a more systematic understanding of pathogenic networks (Figure 1). By consolidating these findings, we provide a holistic framework of MVD pathogenesis, identify critical gaps in current knowledge, and outline future directions for biomarker development and therapeutic targeting.

## Genomics: revealing the genetic basis of MVD

Genomics research, especially genome-wide association studies (GWAS), has made significant achievements in identifying genetic susceptibility to MVD. Studies show that MVP has a clear genetic tendency, existing in both sporadic and familial forms (Table 1) (9). Early linkage analyses had already mapped autosomal dominant MVP to chromosomal regions such as 16p11.2-p12.1 and 11p15.4 (10, 11) and located X-linked myxomatous valvular dystrophy to Xq28 (12) Building on these linkage findings, family-based studies further refined the disease locus to a 2.5-Mb interval and identified a P637Q mutation in *FLNA* as the causal variant for X-linked myxomatous valvular dystrophy (13) Subsequently, in a large multigenerational family with non-syndromic MVP, a missense mutation in *DCHS1* was found to co-segregate with the disease. Functional studies demonstrated that this mutation reduces protein stability, disrupts cellular polarity and migration during valve development, and was validated in animal models, thereby establishing *DCHS1* as a key gene in the pathogenesis of familial MVP (14). In addition, recent family-based studies have identified novel genes that further expand the mechanistic landscape of MVP. Mutations in *DZIP1*, a gene involved in primary cilia function, impair ciliogenesis and disrupt extracellular matrix (ECM) organization during development, leading to progressive myxomatous degeneration (15). Similarly, a gain-of-function mutation in *TLL1*, encoding an ECM-regulating metalloprotease, has been shown to enhance enzymatic activity and promote abnormal ECM remodeling (16). Together, these findings highlight the critical roles of cilia-dependent signaling and ECM homeostasis in MVP pathogenesis.

**Table 1:** Genetic loci and variants associated with mitral valve disease.

**Gene**	**Method**	**Sample**	**Function**	**Diseases**	**Reference**
*FLNA*	Linkage Analysis & Sanger Sequencing	2 large X-linked families	Encodes Filamin A; mutations disrupt the cytoskeleton and TGF-*β* signaling, leading to myxomatous valve degeneration.	X-linked MVP	PMID: 17190868
*DCHS1*	Linkage Analysis & WES	2 families and 106 sporadic cases	Encodes a protocadherin; regulates Planar Cell Polarity and cell alignment during valve development.	MVP	PMID: 26258302
*DZIP1*	Linkage Analysis & WES	1 multigenerational pedigree	Regulates primary cilia formation;	MVP	PMID: 31118289
*TLL1*	WES & WGS	1 three-generation family and 13 cases	Encodes a metalloprotease involved in ECM remodeling	MVP	PMID: 39880331
*TNS1, LMCD1*	GWAS	1,412 cases vs. 2,439 controls	*TNS1* and *LMCD1* are involved in cell proliferation/migration, potentially contributing to mitral valve degeneration.	MVP	PMID: 26301497
*GLIS1, TGFB2, TBX5*	GWAS	434 cases and 4,527 controls	Endothelial-to-mesenchymal transition and migratory programs in heart development	MVP	PMID: 34461747
*SPTBN1, LTBP2, NMB, ALPK3*	GWAS	4,884 cases vs. 434,649 controls	Identified candidate genes for risk stratification; developed polygenic risk score for MVP.	MVP	PMID: 35245370
*PRDM5, ZNF469, COL11A1*	WES	80 cases	Sporadic MVP shows genetic heterogeneity; pathogenic mutations linked to early-onset disease and LV dilation.	MVP	PMID: 40131712

WES, whole-exome sequencing; GWAS, genome-wide association studies; MVP, mitral valve prolapse.

In recent years, larger-scale GWAS studies have further revealed new genetic loci associated with MVP. A landmark meta-analysis of two GWAS studies, involving over 1,412 MVP cases and 2,439 controls, identified the first six genetic risk loci for non-syndromic MVP (17). This study provided functional evidence for *LMCD1* and *TNS1*. *LMCD1* encodes a transcription factor, and knocking down its ortholog in zebrafish led to atrioventricular valve regurgitation. Similarly, knockdown of the *TNS1* ortholog, which encodes a focal adhesion protein involved in cytoskeleton organization, produced a similar phenotype in zebrafish. Further evidence showed that Tns1 knockout mice developed enlarged posterior mitral leaflets. This pioneering research not only uncovered the first risk loci for MVP but also suggested new mechanisms related to cytoskeleton organization and valve morphogenesis in the development of mitral regurgitation. Building on this foundation, a 2019 study by Yu et al. used GWAS-driven gene-set analysis to identify *GLIS1* as a new susceptibility gene for MVP (18). Subsequent research continued to expand the genetic landscape. A large-scale GWAS meta-analysis in 2021 further confirmed the association between genes involved in valve and cardiac development and MVP, including *TGFB2* and *TBX5* that are closely linked to endothelial-to-mesenchymal transition (EndMT) and cell migration (19). More recently, a large GWAS meta-analysis by Roselli et al. (20) involving 4,884 MVP cases and 434,649 controls, identified 14 genome-wide significant loci associated with MVP. Through integrative gene prioritization analyses, the authors highlighted candidate genes including *LMCD1*, *SPTBN1*, *LTBP2*, T*GFB2*, *NMB*, and *ALPK3*, implicating biological pathways related to TGF-*β* signaling and cytoskeletal organization, and extracellular matrix regulation. In addition, the study developed a novel polygenic risk score (PRS) for predicting MVP risk, marking a step towards personalized risk assessment (20).

In addition to GWAS, which primarily identifies common variants, research strategies have expanded to include techniques like whole-exome sequencing (WES) to detect rare variants. Wang Q. et al. employed WES to analyze 80 cases of sporadic MVP in a Southern Chinese population, marking a shift from broad association studies to a deeper investigation of rare genetic variations (21). This study successfully identified novel disease-associated genes (*PRDM5*, *ZNF469*, and *COL11A1)* and variants, providing insights into the genetic heterogeneity of MVP across different populations.

These findings collectively point to the core roles of the cytoskeleton, extracellular matrix homeostasis, and cardiac development regulatory pathways in the pathogenesis of MVD, providing new clues for targeted therapy. Despite these advances, several limitations remain. Most GWAS are predominantly based on European ancestry populations, limiting generalizability. In addition, modest sample sizes in earlier studies may reduce statistical power. Moreover, GWAS are less effective at capturing rare variants and do not directly establish causal mechanisms, underscoring the need for integrative and functional validation studies.

## Epigenomics: connecting genetic information with environmental regulation

As a complex polygenic disease, MVD is influenced by both genetic susceptibility and environmental exposures. Factors such as long-term air pollution exposure (22), environmental noise (22) and other environmental stressors (23) have been suggested to contribute to cardiovascular remodeling and may influence disease susceptibility through epigenetic mechanisms. Early studies in MVD showed that mitral valves exhibit promoter hypermethylation in genes related to lipid metabolism and inflammation, indicating a role for epigenetic regulation in valvular homeostasis (24).

DNA methylation and chromatin accessibility are two core aspects of epigenetic regulation that determine the transcriptional potential of genes. Kyryachenko et al. provided the first genome-wide open chromatin profiles of human mitral valves, identifying specific gene regulatory landscapes in both healthy and diseased states (25). Functional studies further revealed that the *TNS1* gene, identified in the GWAS, is located within one of open chromatin regions and epigenetically regulated. Editing its regulatory region significantly altered *TNS1* expression and cellular behavior, providing direct evidence of the epigenetic regulatory role of this region and its causal link to disease mechanisms (25). Their work linked MVP risk loci to changes in chromatin accessibility, suggesting that genetic susceptibility may be mediated through altered gene regulation. Later, Halawa et al. performed a genome-wide DNA methylation profile analysis on non-diseased human aortic and mitral valves. They found 584 differentially methylated promoters, and the associated genes were enriched in signaling pathways crucial for valve physiology and pathology, such as WNT, Integrin, TGF-*β*, and NOTCH (24). This indicates that even in a healthy state, different valves have unique epigenetic regulation patterns, which may affect their response to pathological stimuli.

Non-coding RNAs (ncRNAs), including microRNAs (miRNAs) and long non-coding RNAs (lncRNAs), are also gaining prominence in MVD research (Table 2**)**. In human studies, *miR-409-3p* and *miR-148b-3p* are significantly downregulated in heart failure patients with mitral regurgitation (MR) (26), while *miR-133a* levels are significantly correlated with echocardiographic parameters and inversely associated with severe secondary MR (sMR) (27). In MVP, *miR-331-3p* is markedly upregulated in MFS patients, while *miR-150-5p* is involved in ECM regulation (28, 29). miRNAs such as *miR-500*, *-3174*, and *-17* also significantly differ between Barlow's disease (BD) and fibroelastic deficiency (FED) (30), highlighting their potential in molecular subtyping. In animal models, *miR-30b-5p* is highly expressed in canine myxomatous mitral valve disease and associated with valvular lesions, and its cross-species expression profile further enhances its biomarker potential (31, 32). In the MVP-affected valves of dogs model, *miR-145* is upregulated in both valve interstitial cells (VICs) and small extracellular vesicles and regulated the KLF4–*α*SMA axis, promoting VIC activation toward a myofibroblast phenotype (33). LncRNA, though less studied, show promising regulatory potential. A total of 127 differentially expressed lncRNAs were identified in MVP patient blood samples (34). These studies indicate that non-coding RNAs are not only potential diagnostic markers but also possible new targets for future therapeutic interventions. While these advances are notable, epigenomic studies in MVD remain limited by several factors. Current investigations are constrained by relatively small sample sizes and limited availability of human mitral valve tissues. Moreover, most studies are cross-sectional in nature, lacking temporal resolution to capture dynamic epigenetic changes during disease progression. In addition, functional validation of identified regulatory elements is still insufficient, which limits the ability to establish causal links between epigenetic alterations and disease mechanisms.

**Table 2:** Potential MVD biomarkers were discovered in epigenomics studies.

**Biomarkers**	**Sample Source**	**Sample Type**	**Function**	**Disease**	**Reference**
miR-409-3p, miR-148b-3p	Human	Serum	Fibrinogen production, induces apoptosis	HF in MR	PMID: 27505319
miR-133a	Human	Venous blood	Activates remodeling/hypertrophic compensation	sMR	PMID: 32780418
miR-30b-5p	Canine	Plasma	Anti-apoptotic and anti-inflammatory	MMVD	PMID: 35816500
miR-331-3p	Human	Venous blood	Associated with myocardial hypertrophy	MFS	PMID: 29530068
miR-150-5p	Human	Venous blood	Signal transduction, tissue remodeling	MFS	PMID: 28679133
miR-500, miR-3174, miR -17	Human	Valve tissue	Distinguishes MMVP from FED	MMVP vs. FED	PMID: 27213335
miR-145	Canine	Small extracellular vesicles	Induces myofibroblast phenotype transition	MMVP	PMID: 38338749

HF, Heart failure; sMR, secondary mitral regurgitation; MMVD, myxomatous mitral valve disease; MFS, Marfan syndrome; FED, fibroelastic deficiency.

## Transcriptomics: from dynamic expression to mechanism analysis

Transcriptomics uses high-throughput sequencing technology to analyze the expression levels of all RNA transcripts in a specific tissue or cell. This provides direct evidence for understanding gene expression changes in a disease state. In recent years, the application of single-cell RNA sequencing (scRNA-seq) technology has elevated the research resolution to the single-cell level, greatly advancing the understanding of cell heterogeneity and pathological mechanisms in MVD.

### Bulk transcriptomics: stage-dependent gene expression and pathway remodeling

Transcriptomic analyses of human valve have been crucial in uncovering molecular pathways involved underlying MVD and in revealing disease stage, and tissue-specific remodeling programs. In MR, temporal transcriptomic patterns observed in human myocardial samples support a transition from an early compensatory phase to a later decompensatory stage. During decompensation, *CTGF* is markedly upregulated and closely associated with extracellular matrix remodeling and fibrotic responses, underscoring its pivotal role in adverse myocardial remodeling (35). In parallel, dysregulation of mitochondrial- and calcium-handling–related genes, such as *SERCA2*, indicates that impaired energy metabolism may further exacerbate functional deterioration as the disease progresses (36).

In contrast to MR, MVP predominantly involves pathological remodeling of valve architecture and ECM homeostasis. Transcriptomic studies of human MVP valve tissues consistently demonstrate upregulation of genes associated with ECM degradation and remodeling (e.g., *MMPs*, *cathepsins*, and *ADAMTSs*), along with activation of the TGF-*β* signaling pathway, identifying it as a central driver of MVP pathogenesis (37, 38). Importantly, distinct molecular signatures have been observed between MVP subtypes. For example, BD and FED exhibit divergent expression profiles, with accumulation of the ADAMTS5 substrate versican in BD but reduced levels in FED, highlighting pronounced molecular heterogeneity underlying clinically defined MVP subtypes (39).

Animal models are indispensable for dissecting causal mechanisms and validating temporal and molecular features suggested by human transcriptomic studies. In a rat model of MR induction, bulk RNA sequencing reveals a clear stage-dependent transcriptional program, in which early MR (0–10 weeks) is characterized by activation of cardiomyocyte remodeling and oxidative stress–related pathways, whereas later stages (20–40 weeks) are dominated by ECM-related pathways (40). This dynamic shift mirrors the compensatory-to-decompensatory transition observed in human myocardium, reinforcing the translational relevance of these models.

Similarly, animal models of MVP provide mechanistic insights into genetically driven and context-dependent transcriptional dysregulation. In Filamin A-mutant rats, transcriptomic alterations are enriched in pathways related to ECM homeostasis, endothelial-to-mesenchymal transition, cell migration, and immune responses, indicating complex interactions between genetic susceptibility and cellular remodeling processes (41). In contrast, Cavalier King Charles Spaniel dogs with spontaneous MVP exhibit upregulation of contractile genes such as *CNTN3* and *MYH1* (42), while Marfan syndrome (MFS) mouse models show predominant enrichment in immune regulation and inflammatory response pathways (37). These interspecies differences further emphasize the heterogeneity of MVP pathobiology and the importance of model selection.

Together, transcriptomic studies have evolved from simple DEG analysis to a comprehensive tool for dissecting disease progression, remodeling dynamics, and molecular subtype classification in MVD.

### Single-cell transcriptomics: cell-type–specific mechanisms and intercellular crosstalk

The application of Single-cell RNA sequencing technology has provided us with a more detailed cellular map of MVD. Recent single-cell studies in mice demonstrated that only a small subset of endocardial cells with specific metabolic features in the atrioventricular canal (AVC) region possess endothelial-to-mesenchymal transition (EndMT) potential. This process is regulated by glycolysis, glutamine metabolism, and primary cilium-related mechanisms (43). Notably, only around 40 endocardial progenitor cells, through precise proliferation, migration, and spatial rearrangement, can give rise to a functional valve structure comprising the fibrosa, spongiosa, and ventricularis layers (43). In parallel, conditional knockout of *Dicer1*, a key enzyme for microRNA biogenesis, has been shown to cause mitral valve dysplasia, characterized by leaflet thickening, stenosis, and functional regurgitation. Single-cell transcriptomic profiling of Dicer1-deficient valves revealed impaired mesenchymal cell maturation, dysregulated expression of ECM-related genes, and abnormal activation of critical transcription factors involved in valve remodeling, such as *Twist1, Sox9*, and *Msx* (44). The immune cells in the mitral valve undergo significant changes during postnatal development. On postnatal day 7, the immune cells in the mitral valve include T cells, dendritic cells, mast cells, and macrophages, with macrophages primarily in the M1 type. By postnatal day 30, the characteristics of immune cells change, with M2 macrophages becoming dominant, and the number of mast cells and T cells decreases, reflecting a shift in the immune system from a naïve state to an effector memory T cell state (45). These findings offer a cellular blueprint for understanding normal valve morphogenesis and congenital malformations.

In disease models, single-cell techniques have also uncovered critical temporal windows. In a murine model of myxomatous MVD, Wnt signaling was aberrantly activated in VICs and valvular endothelial cells (VECs) even before visible structural changes appeared (46). Further intervention studies demonstrated that inhibiting the Wnt pathway at one month of age effectively prevented valve pathology—including ECM abnormalities, leaflet thickening, and macrophage infiltration—whereas interventions after two months showed no significant benefit. This indicates that Wnt signaling primarily drives early-stage disease progression rather than maintaining late-stage pathology. This finding confirms the existence of time-dependent molecular regulation and provides a strategic basis for selecting early intervention biomarkers.

Clinically, scRNA-seq has also been applied to map the cellular landscape of human MR valves. The study found that six major cell types were primarily identified in normal human mitral valves, including VICs, myeloid cells, lymphocytes, VECs, mast cells, and myofibroblasts (47, 48). Notably, a subset of proliferative endothelial cells expressing FABP4 was significantly reduced in MR valves. This subpopulation is thought to represent the “progenitor” cells of VECs, and their depletion may reflect impaired repair capacity and accelerated disease progression (48). This study highlights a critical concept that not only are functional changes in VICs tightly linked to structural remodeling, but the exhaustion of VECs may also be a key driver of disease advancement. Therefore, future therapeutic strategies targeting the imbalance in VEC-VIC interactions may offer greater intervention potential than conventional anti-fibrotic approaches.

Overall, the application of single-cell technologies continues to expand our understanding of mitral valve development and disease. Although current human scRNA-seq data on the mitral valve remains limited, the future integration of spatial transcriptomics, single-cell multi-omics, and temporal sampling technologies is expected to yield a comprehensive, cell-based, spatially-resolved atlas of the mitral valve across its developmental and pathological continuum.

### Spatial transcriptomics: mapping cellular niches and regional gene programs

Spatial transcriptomics has recently emerged as a powerful technology that enables the mapping of gene expression within intact tissue architecture, thereby linking transcriptional programs with their spatial microenvironment. Compared with conventional bulk or single-cell RNA sequencing, spatial transcriptomics provides unique insights into the spatial organization of cell populations and signaling niches within complex tissues. Recent studies have applied this approach to the mitral valve, characterizing specialized cell populations during early human development. Atrial-side valvular endothelial cells were enriched for WNT signaling genes (*WNT2, WNT4*), while ventricular-side cells expressed BMP genes (*BMP4, BMP6*), detectable as early as six weeks of gestation, suggesting hemodynamic forces shape early valve patterning. The valve also contains interstitial cells and two mesenchymal populations, one highly expressing *PENK*, indicating a potential neural crest contribution (49). Genes linked to mitral valve prolapse provide further mechanistic insights. *DCHS1*, an atypical cadherin in the planar cell polarity pathway, was studied using a Dchs1-HA knock-in mouse. *DCHS1* was restricted to non-cardiomyocyte lineages, dynamically localized during development, and later formed polarized extensions connecting endothelial and neighboring cells, implying heterotypic and homotypic interactions. Proteolytic cleavage generated an intracellular fragment, and co-expression with F*AT4* suggests a conserved non-myocyte DCHS1–FAT4 signaling axis mediating intercellular communication (50). Direct spatial transcriptomic profiling of mitral valve tissues in disease remains limited. One study of the inferobasal left ventricle in mitral valve prolapse patients revealed region-specific transcriptional changes, including localized *NPPB* upregulation, reflecting stretch-induced remodeling. Although valve leaflets were not directly analyzed, this highlights the potential of spatial transcriptomics to investigate mitral valve–associated tissue remodeling (51).

While recent transcriptomic studies have provided important insights, several limitations remain. Many analyses rely on end-stage surgical samples, limiting relevance to early disease stages, and sample sizes, particularly in human single-cell datasets, are often modest, affecting robustness. In addition, cross-species differences between human tissues and animal models may complicate interpretation and translation. These limitations highlight the potential of spatially resolved transcriptomic approaches to complement single-cell analyses and to uncover spatially restricted molecular programs involved in valve-associated cardiac remodeling. As spatial transcriptomic technologies continue to evolve, their application to human mitral valve tissues may provide new insights into microenvironment-specific signaling and disease heterogeneity.

## Proteomics: revealing molecular markers of mitral valve disease

Proteomics has emerged as a vital tool in mitral valve disease research (Table 3). An early plasma proteomic study of asymptomatic patients with isolated moderate-to-severe MR demonstrated significantly decreased levels of haptoglobin, platelet basic protein, and complement component C4b (52), suggesting early alterations in systemic immune and oxidative stress–related pathways. Another subsequent serum proteomic cohort study demonstrated that metabolic proteins such as high-density lipoprotein and apolipoprotein A1 declined progressively with MR severity, with apolipoprotein A1 identifying as an independent predictor of disease progression (53). Additionally, elevated levels of autophagy-related protein LC3 in valve tissue suggested activation of stress responses and repair mechanisms at early disease stages. Moreover, recent evidence further links procedural failure in mitral valve repair to the upregulation of specific inflammatory and fibrotic biomarkers (e.g., IL2RA, IGFBP2) in plasma samples, which independently predicted failure and rising pulmonary artery pressure (54).

**Table 3:** Potential MVD biomarkers identified in proteomics studies.

**Proteins**	**Sample Source**	**Sample Type**	**Platform**	** Findings**	**Function**	**Disease**	**Reference**
Haptoglobin, PBP, C4b	Human	Plasma	MS/MS	Upregulated	Hemolysis, platelet dysfunction, complement activation	MVP/MR	PMID: 24140280
Apolipoprotein A1	Human	Serum/plasma	Multiplex MAP	Decreased	Disrupted lipid metabolism/autophagy	MR	PMID: 26405438
IGFBP2, IL2RA	Human	Plasma	Olink Proseek® Multiplex	Upregulated	Immune regulation	post–M-TEER MR	PMID: 40728736
SERPINH1	Canine	Plasma	LC-MS/MS	Upregulated	Collagen production	MMVD	PMID: 37146676
clusterin, peptidase D	Canine	Serum	Nano-LC-MS	Upregulated clusterin, Decreased peptidase D	Anti-inflammatory, ECM activity	MMVD	PMID: 37108311

PBP, platelet basic protein; C4b, mplement component C4b MVP, mitral valve prolapse; MR, secondary mitral regurgitation; MMVD, myxomatous mitral valve disease; ECM, extracellular matrix; M-TEER, mitral valve transcatheter edge-to-edge repair.

Beyond circulating biomarkers, mitral valve tissue proteomic profiling has provided mechanistic insights. In ischemic MR (IMR), MVs from patients with more severe MR showed significant upregulation of proteins related to proteolysis, inflammatory mediators, and oxidative stress, supporting the view that that valve pathology is not merely mechanical but rather driven by active molecular processes (55). In parallel, myocardial tissue proteomic studies in MR patients further revealed ECM remodeling and cytoskeletal upregulation, along with downregulation of metabolic proteins, implying that MR may exacerbate ventricular structural changes and metabolic dysregulation, contributing to heart failure (56). Proteomic analysis of mechanically stretched, endothelium-denuded valve leaflets revealed downregulation of glycolysis- and oxidoreductase-related proteins in VECs, indicating a metabolic shift toward a low-activity state (57).

Sex-based molecular differences have also been observed. Female MR patients exhibited less pronounced ECM remodeling and preserved metabolic profiles, correlation with milder ventricular remodeling and better cardiac function. These findings align with clinical data suggesting sex-based differences in MR progression and outcomes (56).

Beyond human studies, animal models, particularly the canine model of MMVD, have established early-stage MVD proteomic profiles. Proteomic profiling of MMVD tissues identified over 100 differentially expressed proteins, many of which were altered even in preclinical stages (58, 59). Advanced LC-MS TMT-based quantitative analyses of serum samples identified 21 stage-specific markers, including clusterin and peptidase D (60). (Serum) Additionally, the fibrosis-related protein SERPINH1 was significantly elevated in plasma samples at stage B2, underscoring its potential as a diagnostic indicator (60, 61).

Despite these advances, challenges remain. Current proteomic studies are often limited by small sample sizes, lack of standardized sample acquisition protocols, and insufficient validation of candidate proteins. Many functional interpretations still depend on transcriptomic correlation, underlining the need for more integrative, mechanistically focused investigations.

## Metabolomics: measurement of molecular phenotypes

Metabolomics has emerged as a powerful approach for studying MVD, serving as a bridge between gene expression and phenotypic outcomes. In human plasma-based metabolomic studies, metabolic dysregulation has been observed in both MR and mitral stenosis (MS), particularly in pathways involving energy metabolism, amino acid cycles, calcium homeostasis, and inflammatory responses. Nuclear magnetic resonance (NMR) based profiling identified reduced levels of formate and lactate, indicating a remodeling of metabolic pathways and suggesting their potential as biomarkers for early disease classification (62).

In terms of targeted metabolomics, advanced targeted plasma metabolomics analyses combined with untargeted eicosanoid profiling have shown that in patients with secondary MR complicating heart failure with reduced ejection fraction, six eicosanoids were significantly up-regulated, whereas other metabolites did not differ significantly (63).

Beyond targeted studies, untargeted metabolomics has revealed widespread systemic metabolic alterations associated with valvular disease. Serum-based untargeted metabolomic analyses have revealed the interplay between valvular disease and systemic organ function. Impaired renal function was found to disrupt the glutathione cycle and fatty acid oxidation, exacerbating disease progression and supporting the “heart-kidney-gut axis” as a novel regulatory network in MVD development (64). Additionally, plasma-based untargeted metabolomics identified distinct metabolic signatures in MVD-associated atrial fibrillation, including alterations in galactose metabolism, unsaturated fatty acid synthesis, and linoleic acid metabolism, underscoring the potential for metabolomics to guide comorbidity management (65).

Animal models further substantiate the role of metabolic alterations in MVD. In canine degenerative mitral valve disease, serum-based untargeted metabolomics revealed substantial changes in fatty acid and glucose metabolism as well as oxidative stress pathways (66). Importantly, 94 metabolites were significantly altered during the preclinical phase when the cardiac murmur first appeared, highlighting the value of metabolites as early warning signals (64). Interestingly, nutritional interventions, such as supplementation with DHA and EPA, were shown to reduce levels of glycerophosphocholine and xanthine, while increasing lactate and furaneol—an antioxidant metabolite—in serum-based untargeted metabolomic analyses, providing a potential role for dietary modulation in slowing valvular disease progression (67). As MVD progresses toward heart failure, significant remodeling of lipid metabolic profiles was observed in untargeted serum metabolomic studies, presenting a typical “heart failure-type metabolic fingerprint” (68). In more severe cases, untargeted serum metabolomics further revealed elevated levels of medium- and long-chain dicarboxylic acids and their acylcarnitines, reflecting extreme metabolic exhaustion (69).

Metabolomics sheds light on the systemic and valvular-specific metabolic shifts underlying MVD, the future integration with genomics and proteomics may further clarify the causal links between metabolic remodeling and valvular degeneration. However, metabolomic studies in MVD also face several limitations. Metabolic profiles are highly sensitive to systemic factors such as comorbidities, diet, and medication use, which may confound valve-specific signals. In addition, most studies are based on relatively small, cross-sectional cohorts, limiting statistical power and the ability to infer temporal changes. These limitations may obscure valve-specific metabolic signatures and reduce reproducibility across studies.

## Machine learning: applications from screening to clinical decision-making

In addition to omics technologies, machine learning has shown considerable potential in the screening, diagnostic evaluation, and risk prediction of mitral valve disease. For early screening, machine learning models can utilize simple clinical information or low-cost examinations for preliminary identification. For example, a random forest model developed using clinical history, physical examination findings, and quality-of-life questionnaire data was able to stage canine myxomatous mitral valve disease and effectively distinguish asymptomatic from clinical stages (70). In addition, a recurrent neural network–based algorithm can detect and grade heart murmurs from electronic stethoscope recordings, thereby assisting in the identification of MMVD and its preclinical stages (71).

In imaging analysis, deep learning has been widely applied to the automated interpretation and quantitative assessment of echocardiography. Convolutional neural networks can automatically identify echocardiographic views and detect MR (72), while an Attention-UNet–based segmentation model can automatically extract key structures of the mitral leaflets and annulus to derive morphological indicators associated with mitral valve prolapse or stenosis (73). At the diagnostic level, the deep learning model DROID-MVP can accurately identify mitral valve prolapse by analyzing large-scale echocardiographic videos, achieving an external validation AUROC of over 0.96, indicating strong clinical application potential.

For disease severity assessment, machine learning models can automatically integrate multiple echocardiographic parameters to grade MR. For instance, a fully automated multiparametric machine learning system can rapidly determine MR severity by analyzing 16 echocardiographic parameters and accurately identify moderate-to-severe MR (74). Another end-to-end deep learning system directly analyzes complete echocardiographic studies and color Doppler videos, demonstrating high agreement in MR severity classification in both internal and external datasets (AUC ≈ 0.98) (75). Furthermore, the large-scale AI system DELINEATE-Regurgitation can automatically classify multiple types of valvular regurgitation (AR, MR, and TR) and further predict the risk of MR progression (76).

Beyond diagnosis, machine learning has also been applied to prognostic evaluation and treatment decision support. For example, a random forest model integrating echocardiographic, radiographic, and laboratory variables can predict the risk of heart failure in dogs with MMVD (77). In human studies, machine learning models have been used to predict the occurrence of moderate-to-severe MR after transcatheter aortic valve replacement and identify key clinical and echocardiographic predictors (78). In addition, a support vector machine model based on preoperative echocardiographic data has been developed to assess the complexity and risk of failure in mitral valve repair surgery, providing guidance for clinical surgical planning (79).

Overall, machine learning demonstrates broad potential across multiple stages of mitral valve disease management. From primary screening and automated imaging analysis to disease severity grading and prognostic prediction, these approaches may improve diagnostic efficiency and objectivity while supporting more precise and individualized treatment strategies. While machine learning approaches are increasingly applied in MVD research, they remain limited by small and heterogeneous datasets, potential overfitting, lack of standardized validation, and limited interpretability, which may hinder clinical translation. These challenges raise concerns about the generalizability and real-world applicability of current machine learning models. In the future, integrating machine learning with multi-omics data may further enhance the identification of molecular signatures and improve precision medicine strategies in MVD.

## Multi-Omics integrated analysis: an integrated framework for decoding MVD

MVD involves multiple cell types, signaling pathways, and dynamic pathological changes, making it difficult to fully capture with single-omics approaches. Multi-omics integration of genomics, epigenomics, transcriptomics, proteomics, and metabolomics enables the systematic dissection of the molecular mechanisms underlying MVD (20). This allows for a deeper understanding of the complex causal relationships from gene to phenotype. The rapid development of multi-omics technologies has generated large-scale datasets that require specialized computational tools for data processing, statistical analysis, and biological interpretation. A wide range of bioinformatics programs has therefore been developed to support analyses across different omics layers.

The strategies for multi-omics integration have evolved from simple data concatenation to sophisticated representation learning, focusing on capturing the intricate inter-layer correlations. For instance, MOFA (80) and iClusterPlus (81) employ joint matrix factorization to decompose variations across genomics, epigenomics, and transcriptomics into latent factors. For targeted biomarker discovery, supervised frameworks like mixOmics (DIABLO) (82) leverage sparse Partial Least Squares to pinpoint key variables in proteomics and metabolomics, while network-based tools like Similarity Network Fusion (SNF) (83) reveal hidden disease subtypes by merging modality-specific patient similarity graphs.

Beyond these classical approaches, deep generative models have demonstrated superior performance in denoising high-dimensional data and uncovering non-linear regulatory mechanisms. Variational Autoencoder (VAE)-based frameworks, such as OmiVAE (84), MMD-VAE (85), and OmiEmbed (86), facilitate joint embedding and multi-task learning by processing gene expression, DNA methylation, and miRNA data within unified architectures. Furthermore, omicsGAN (87) utilizes Generative Adversarial Networks to integrate mRNA and miRNA expression alongside their interaction networks, while advanced frameworks like MOSA (88) employ conditional multi-modal VAEs to synthesize data across proteomics, metabolomics, and CRISPR-Cas9 screens. Collectively, these computational tools provide the necessary rigor to decode the multi-layered molecular landscapes of disease. Representative computational tools commonly used in these omics studies, together with their potential applications in mitral valve disease research, are summarized (Table 4).

**Table 4:** Computational tools commonly used in multi-omics studies of mitral valve disease.

**Omics layer**	**Computational Tool**	**Primary Function**	**Potential Applications in MVD Research**	**Reference**
Genomics	PLINK	Genome-wide association analysis, genotype QC	Identification of genetic loci associated with disease phenotypes	PMID: 17701901
Genomics	METAL	Meta-analysis of GWAS datasets	Integration of multi-cohort genetic studies	PMID: 20616382
Genomics	GCTA	Genetic heritability estimation & complex trait analysis	Estimation of heritability and genetic architecture	PMID: 21167468
Genomics	FUMA	Functional mapping and annotation of GWAS results	Prioritization of candidate genes/regulatory elements	PMID: 29184056
Transcriptomics	STAR	RNA-seq read alignment	Processing transcriptomic data	PMID: 23104886
Transcriptomics	HISAT2	RNA-seq alignment & transcript assembly	Gene expression profiling	PMID: 31375807
Transcriptomics	DESeq2	Differential expression analysis	Identification of dysregulated genes	PMID: 25516281
Transcriptomics	edgeR	Differential expression for RNA-seq	Detection of transcriptional changes	PMID: 19910308
Transcriptomics	Seurat	Single-cell RNA-seq analysis & clustering	Cellular heterogeneity characterization	PMID: 29608179
Transcriptomics	Monocle	Pseudotime trajectory analysis	Cell differentiation and development pathways	PMID: 24658644
Proteomics	MaxQuant	Protein identification & label-free quantification	Identification of protein biomarkers	PMID: 19029910
Proteomics	Perseus	Statistical analysis & visualization	Differential proteins and enriched pathways	PMID: 27348712
Proteomics	STRING	PPI network analysis	Molecular interaction networks	PMID: 33237311
Metabolomics	XCMS	Peak detection & feature extraction from LC-MS	Metabolic feature identification	PMID: 16448051
Metabolomics	MZmine	Metabolite peak detection & quantification	Untargeted metabolomics processing	PMID: 20650010
Metabolomics	MetaboAnalyst	Statistical analysis & pathway enrichment	Altered metabolic pathways in disease	PMID: 34019663
Multi-omics	MOFA	Bayesian Group Factor Analysis	Identifying shared and unique latent drivers	PMID: 29925568
Multi-omics	mixOmics (DIABLO)	Sparse Partial Least Squares	Supervised integration and biomarker discovery across multiple omics layers	PMID: 30657866
Multi-omics	SNF	Similarity Network Fusion	Uncovering hidden disease subtypes by merging modality-specific patient similarity graphs	PMID: 24464287
Multi-omics	iClusterPlus	Latent variable modeling	Integrative clustering and molecular stratification of disease cohorts	PMID: 23431203
Multi-omics	OmiVAE	Supervised Variational Autoencoder with SHAP	Feature extraction, disease classification, and interpretable biological signal detection	PMID: 34402865
Multi-omics	MMD-VAE	VAE with MMD regularization	Joint embedding and survival analysis using DNAm, CNV, and transcriptomics	PMID: 33737557
Multi-omics	OmiEmbed	Multi-task Variational Autoencoder	Unified dimensionality reduction for regression, classification, and survival prediction	PMID: 34207255
Multi-omics	omicsGAN	Generative Adversarial Networks	Integrating based on regulatory interaction networks	PMID: 34415323
Multi-omics	MOSA	Conditional MVAE with contrastive loss	Synthesizing data or drug discovery	PMID: 39614072

Recent studies further illustrate how integration of the multiple omics layers can help connect genetic variation, transcriptional regulation, and cellular responses involved in mitral valve disease. In a FilaminA-P637Q knock-in rat model of MVD, integrative RNA sequencing and ATAC-seq analyses of mitral valve tissues identified dysregulation in extracellular matrix homeostasis, endothelial-to-mesenchymal transition, chemotaxis, and immune-related pathways. Notably, chromatin accessibility analysis highlighted TGF-*β* signaling and inflammatory responses as central regulatory mechanisms in early valve dysplasia (41). In human MVP studies, integrative analyses combining epigenomic annotations, transcriptomic quantitative trait loci, and proteomic data identified candidate genes located within novel genetic loci, including *LTBP2, TGFB2, LMCD1*, and *SPTBN1,* and emphasized the involvement of TGF-*β* signaling, cytoskeletal organization, and overlap with cardiomyopathy-related genes such as *ALPK3* (20). Beyond structural remodeling, immune regulation has also been investigated using multi-omics strategies. Through integrative Mendelian randomization, circulating proteomics, and transcriptomic immune profiling, 33 immune-related blood proteins were identified as causally associated with MVP risk, highlighting macrophage polarization and adaptive immune regulation as important contributors to disease progression (89). In MVP patients, dysregulated expression of RNA-binding proteins and alternative splicing events were associated with immune activation and apoptosis (90), highlighting RNA processing as a potential pathological mechanism and therapeutic target.

In a canine model of degenerative mitral valve disease (DMVD), combined metabolomics and transcriptomics showed enhanced glycolysis (e.g., *GLUT3/6* upregulation) and suppressed fatty acid oxidation (e.g., *ACSL1* downregulation) (66), suggesting that metabolic shifts may contribute to early disease initiation rather than being a late consequence. Beyond these mechanistic insights, Chen et al. integrated GWAS, expression quantitative trait loci (eQTL), methylation quantitative trait loci (mQTL), and protein quantitative trait loci (pQTL) data together with multiple genetic analysis methods such as Mendelian randomization (MR) and colocalization analyses. This integrative analysis identified Neuromedin B (*NMB*) as a potential druggable gene for MVP and proposed the flavonoid quercetin as a promising therapeutic candidate based on multi-omics analyses and computational validation (91). Furthermore, combined proteomic and metabolomic analyses in MVD patients with atrial fibrillation revealed lipid metabolic reprogramming and suppression of the PPAR*α* pathway, indicating close interactions between metabolic remodeling and disease progression (65).

Overall, multi-omics integration has begun to provide complementary insights into the genetic, transcriptional, immune, and metabolic alterations observed in MVD. However, most studies to date rely on partial data integration and relatively small or heterogeneous cohorts. Future studies combining multi-omics data with longitudinal clinical phenotyping and functional validation will be necessary to fully elucidate causal mechanisms and translate these findings into clinical applications.

## Concluding remarks and future perspectives

Over the past decade, omics technologies, centered on high-throughput sequencing, have greatly advanced our understanding of the molecular mechanisms of mitral valve disease. Genomics research has revealed a large number of genetic loci and candidate genes associated with MVD, providing a basis for risk prediction and family screening. Epigenomics and transcriptomics research have clarified the complexity of gene expression regulation in the pathological process of MVD, especially the roles of DNA methylation, histone modifications, and non-coding RNAs. Proteomics has not only deepened the understanding of ECM remodeling in valve tissue but has also made important progress in finding circulating biomarkers. Multi-omics integrated analysis, by connecting different molecular levels, has built a complete knowledge chain for us, from genetic variation to functional dysregulation to clinical phenotype.

Importantly, these advances have several key implications for the understanding and management of MVD. First, multi-omics studies have substantially improved our understanding of the molecular mechanisms underlying valve degeneration, particularly those involving extracellular matrix remodeling, inflammatory signaling, endothelial-to-mesenchymal transition, cytoskeletal organization, and metabolic reprogramming. These mechanistic insights help explain how genetic susceptibility, environmental factors, and cellular responses collectively contribute to progressive structural and functional alterations of the mitral valve. Second, the identification of circulating RNAs, proteins, and metabolites has opened new opportunities for the development of biomarkers that may enable earlier diagnosis, disease monitoring, and risk stratification. Such molecular indicators may complement traditional imaging approaches and provide non-invasive tools for detecting early pathological changes before overt structural abnormalities become apparent. Third, omics-based molecular profiling has revealed significant heterogeneity within clinically defined entities such as MVP, providing a basis for refined disease subtyping and improved characterization of disease progression. This molecular stratification may help explain differences in clinical outcomes and treatment responses among patients who share similar anatomical diagnoses. Finally, integrative analyses across genomic, transcriptomic, proteomic, and metabolomic layers have helped prioritize candidate genes and pathways that may serve as potential therapeutic targets. By linking genetic variation to downstream molecular and cellular alterations, these approaches provide a rational framework for identifying druggable pathways and guiding the development of disease-modifying therapies for MVD.

Despite significant progress, omics research on MVD still faces challenges. First, most studies have relatively small sample sizes. Larger-scale, multi-center prospective cohort studies are needed to validate the discovered markers and targets. Second, current research mostly focuses on late-stage valve tissues with significant pathological changes. Little is known about the molecular events of disease initiation and early progression. In the future, using single-cell multi-omics technologies to study early-stage diseased tissues will help capture key initiating signals. In addition, combining omics data with advanced computational biology and artificial intelligence methods can more effectively mine valuable biological information and predictive models from massive data. Finally, strengthening translational research from animal models to human clinical samples and conducting clinical trials of targeted drugs based on omics discoveries are key steps to translate basic research findings into clinical practice. It is foreseeable that with the continuous improvement of technology and the deepening of research, omics-based precision medicine will bring revolutionary breakthroughs to the diagnosis, prevention, and treatment of mitral valve disease in the near future.

In conclusion, omics integration has significantly advanced our understanding of mitral valve disease complexity. By linking molecular alterations to clinical phenotypes, these approaches offer opportunities to redefine disease classification, identify therapeutic targets, and develop precision medicine strategies. Continued technological innovation and rigorous validation will be essential to fully realize the potential of omics in transforming mitral valve disease management.
